# Photobiomodulation therapy and postoperative recovery after abdominoplasty: a non-randomized pilot study

**DOI:** 10.1007/s10103-026-05007-y

**Published:** 2026-08-22

**Authors:** Carmen Lucia Kretiska Araujo, Graziele Silveira Fardin, Ana Paula Bernardi, Joane Severo Ribeiro, Ricardo Vitiello Schramm, Isadora Frois Ourique, Maria Luiza Santos, Betina Vescovi, Tássio Fernando Crusius, Flávia Marafon, Denis Valente, Níveo Steffen, Patricia Viana da Rosa, Alessandra Peres

**Affiliations:** 1 Universidade Federal de Ciências da Saúde de Porto Alegre, Porto Alegre, Brazil; 2 Universidade Federal de Jataí, Jatai, Brazil

**Keywords:** Photobiomodulation therapy, Low-level laser therapy (LLLT), Abdominoplasty, Oxidative stress, Cytokines, Postoperative pain

## Abstract

Photobiomodulation (PBM) has been proposed as a non-invasive therapeutic strategy to support wound healing and modulate oxidative and inflammatory responses. However, clinical data evaluating its effects in the postoperative period of abdominoplasty remain limited. This non-randomized clinical trial included 31 female patients undergoing abdominoplasty, divided into two groups: PBM intervention (*n* = 15) and control (*n* = 16). Salivary samples were collected at three time points: preoperative (T0), 48 h postoperative (T1), and 7 days postoperative (T2). Inflammatory markers (TNF-α, IL-10), oxidative stress indicators (TBARS, nitrites), and pain scores were assessed. PBM was applied once using an 808 nm infrared laser (100 mW, 3 J/point) before hospital discharge. Statistical analyses included the Shapiro-Wilk test, Friedman test, Mann–Whitney U test, Spearman’s correlation, and multiple linear regression. PBM did not produce statistically significant changes in TNF-α, IL-10, nitrites, or pain scores compared to the control group. However, a statistically significant reduction in TBARS levels was observed at 48 h postoperatively in the unadjusted analysis (*p* = 0.040), although this effect was not maintained after adjustment for baseline variability. Physical activity was associated with lower nitrite concentrations (*p* = 0.047), indicating a modulatory interaction between exercise and nitric oxide metabolism. Although a single session of PBM did not significantly alter most inflammatory and oxidative markers, the intervention was well tolerated during the postoperative period. These preliminary findings support the safety of PBM during postoperative recovery after abdominoplasty, although larger randomized trials are required to confirm its potential clinical benefits. Limitations include small sample size, single-session PBM, lack of sham control, and no correction for multiple comparisons. Further randomized studies with larger sample sizes and multiple applications are warranted to better elucidate its therapeutic potential.

## Introduction

Surgical procedures such as abdominoplasty trigger complex inflammatory and oxidative responses that influence postoperative recovery and tissue repair. Wound healing is a complex biological process involving the coordinated action of inflammatory cells, epidermal and dermal cells, extracellular matrix components, blood vessels, and plasma-derived proteins, all regulated by cytokines and growth factors [10]. This physiological process is essential for tissue repair following injury or surgical procedures. However, wound healing becomes more challenging in the presence of inflammatory complications, especially after high-complexity procedures such as abdominoplasty.

The inflammatory response triggered by surgical trauma is mediated by a complex network of cytokines that regulate immune activation and tissue repair. Among these mediators, tumor necrosis factor alpha (TNF-α) is a key pro-inflammatory cytokine involved in the early phase of wound healing, promoting leukocyte recruitment, endothelial activation, and amplification of the inflammatory cascade. Conversely, interleukin-10 (IL-10) plays a central anti-inflammatory role by limiting excessive immune responses and promoting the resolution phase of inflammation, thereby contributing to tissue repair and scar modulation. The balance between pro- and anti-inflammatory cytokines is an important indicator of postoperative inflammatory status and recovery dynamics. TNF-α reflects inflammatory activation, whereas IL-10 represents regulatory immune responses. For this reason, both cytokines are widely used as biomarkers to monitor inflammatory processes in clinical and translational studies of tissue injury and wound healing [4, 11, 20, 35].

In addition to inflammatory mediators, oxidative stress plays an important role in tissue injury and postoperative recovery. Thiobarbituric acid reactive substances (TBARS) are widely used as an indirect marker of lipid peroxidation, reflecting oxidative damage to cell membranes caused by reactive oxygen species (ROS). Increased TBARS levels have been associated with surgical trauma, inflammation, and impaired tissue repair. Therefore, TBARS is widely used as a biomarker of oxidative stress in clinical and experimental studies. Nitrite levels were also evaluated as an indirect indicator of nitric oxide (NO) metabolism. Nitric oxide is rapidly converted into more stable metabolites, such as nitrite and nitrate, in biological fluids. Therefore, nitrite concentration is commonly used as an indirect marker of NO bioavailability and endothelial function. Together, TBARS and nitrite measurements provide complementary information about oxidative stress and nitric oxide–related pathways involved in postoperative tissue repair and inflammatory regulation [3, 5, 21].

Saliva has emerged as a valuable biological fluid for monitoring inflammatory and oxidative biomarkers in clinical research. Several studies have demonstrated that salivary cytokines may reflect systemic inflammatory responses, as many circulating mediators can diffuse from blood into saliva through transcellular or paracellular pathways in the salivary glands [20, 34]. Because saliva collection is non-invasive, painless, and easily repeatable, saliva represents a practical alternative to blood sampling for longitudinal monitoring of inflammatory processes, particularly in postoperative and outpatient settings [17].

Photobiomodulation (PBM) has emerged as a promising, non-invasive therapeutic strategy that uses low-level light to modulate cellular processes and accelerate wound healing [18]. PBM interacts with mitochondrial components, particularly cytochrome c oxidase. This interaction increases ATP synthesis, promotes tissue repair, and activates cellular signaling pathways involved in regeneration [28, 37]. PBM has been shown to stimulate fibroblast proliferation and migration. It also improves extracellular matrix deposition and enhances collagen production, processes that are essential for effective wound healing. These effects are particularly relevant in pathological conditions such as diabetic wounds, where fibroblast function is often impaired [16].

Additionally, PBM can regulate gene expression during the healing process. Studies have shown downregulation of matrix metalloproteinases (MMP2 and MMP9) and pro-inflammatory cytokines such as IL-6 and IL-10. At the same time, PBM may upregulate genes such as DNMT3A and bFGF, which are associated with improved wound healing outcomes. Pilar et al., [25]. Clinical trials have also shown that PBM improves scar quality during the early stages of healing when compared to untreated scars, although its long-term effects appear to diminish after one year [29].

Despite these encouraging findings, the efficacy of PBM depends strongly on treatment parameters such as light source, wavelength, energy density, pulse structure, and application duration [18]. The effects of PBM in the acute postoperative period, particularly on inflammatory and oxidative markers in surgical patients, remain poorly explored.

Abdominoplasty represents a relevant clinical model for studying postoperative inflammation and oxidative stress because the procedure involves extensive tissue manipulation, vascular disruption, and a pronounced inflammatory response during the early healing phase.

Given the limited clinical evidence regarding PBM effects on inflammatory and oxidative biomarkers in abdominal surgery, this pilot study was designed to explore potential biological responses during the early postoperative period. Therefore, the present study aimed to investigate whether a single PBM session could modulate inflammatory and oxidative stress responses during the acute healing phase following abdominoplasty. These effects were evaluated through salivary biomarkers of inflammation (TNF-α and IL-10) and oxidative stress (TBARS and nitrites). These biomarkers provide complementary information on inflammatory activation and immune regulation during postoperative recovery. Saliva represents a non-invasive and easily accessible biological matrix for monitoring inflammatory and oxidative processes in surgical patients, making it particularly suitable for longitudinal postoperative studies.

## Methods

### Study design and ethical considerations

The study protocol was approved by the Institutional Ethics Committee (approval number 6.308.240) and conducted in accordance with the Declaration of Helsinki. Written informed consent was obtained from all participants. Random allocation was not performed due to the exploratory nature of this pilot study and logistical constraints related to surgical scheduling. This design may introduce allocation bias, which should be addressed in future randomized controlled trials. Both groups were followed using the same assessment schedule. Saliva samples and pain evaluations were performed at three predefined time points: T0 (1–6 h before surgery), T1 (48 h postoperatively), and T2 (7 days postoperatively). These assessments were conducted for both the PBM and control groups to allow longitudinal comparisons of inflammatory and oxidative stress biomarkers as well as postoperative pain outcomes. As a pilot study, no formal sample size calculation was performed. The sample size was determined based on feasibility considerations, participant availability, and the exploratory nature of the study. The primary objective was to generate preliminary data and estimate effect sizes to support the design of future randomized controlled trials.

The selection of assessment time points was based on key phases of the postoperative inflammatory and healing response. The baseline measurement (T0) was obtained 1–6 h before surgery to capture preoperative biomarker levels without surgical influence. The 48-hour postoperative assessment (T1) was chosen because the early inflammatory response and oxidative stress typically peak within the first two days following surgical trauma. The 7-day assessment (T2) corresponds to the transition between the inflammatory and early proliferative phases of wound healing, when initial tissue repair and symptom improvement are expected.

### Participants and group allocation

Eligible participants were women aged 18 years or older scheduled for elective abdominoplasty. Exclusion criteria included acute inflammatory or infectious conditions, use of photosensitizing medications, HIV infection, pregnancy, lactation, and tattoos on the abdominal region.

Participants were allocated into two groups: the intervention group (PBM group), which received photobiomodulation after surgery, and the control group, which received standard postoperative care without PBM. All participants received the same postoperative physiotherapy instructions, including posture adjustments and wound care. The study was not prospectively registered, which limits transparency. Future studies will be prospectively registered to strengthen methodological rigor.

### Photobiomodulation protocol

A single PBM session was applied to investigate the immediate postoperative biological response and to reflect the clinical feasibility of PBM use prior to hospital discharge. PBM was applied using an 808 nm infrared laser device (Elight IRL, DMC, São Carlos, Brazil) in cluster mode, with continuous emission and the applicator positioned perpendicular to the abdominal skin at a distance of 1 cm. Each of the four infrared laser diodes delivered an output power of 100 mW for 30 s, corresponding to 3 J per irradiation site. During each application, the four infrared diodes were activated simultaneously, resulting in a total cluster output power of 400 mW and an energy delivery of 12 J per cluster position. The energy density was calculated using a beam area of 2.26 cm² per irradiation site, resulting in 1.33 J/cm². This beam area was obtained from the optical specifications of the device and was used for all dosimetric calculations.

According to the manufacturer’s specifications, the wavelength tolerance was ± 10 nm and the output power variability was ± 20% of the nominal value. These values represent the operational tolerances of the device and do not indicate equipment malfunction. The device contains four red laser emitters and four infrared laser emitters. In the present study, only the four infrared emitters were activated, operating simultaneously at a nominal wavelength of 808 nm (± 10 nm).

A custom template based on the anatomical vascular zones described by Huger (1979) was developed to standardize the irradiation area. The template encompassed a 36 × 9 cm anatomical region and contained 144 predefined irradiation sites spaced 1 cm apart, with 72 sites per hemibody. The sites were aligned with anatomical landmarks, including the iliac crests and costal margins. A dermatographic pencil was used to transfer these reference points to the skin surface, ensuring reproducible positioning of the laser applicator throughout the procedure. The marked sites did not correspond to holes, perforations, or openings in the template; rather, they represented predefined reference locations used to standardize PBM application.

PBM was applied sequentially, with the cluster positioned for 30 s at each treatment location. During each application, the four infrared laser diodes emitted simultaneously, delivering 3 J to each of four predefined irradiation sites. Therefore, although 144 irradiation sites were marked on the skin, only 36 sequential cluster positions were required because four sites were treated simultaneously at each position. The applicator was repositioned according to a predefined sequence to ensure that all 144 marked sites were irradiated once, without repeating any marked irradiation site during successive applications. Consequently, the total irradiation time was approximately 18 min (36 positions × 30 s). The total energy delivered to the treated area was 432 J (144 irradiation sites × 3 J per site). The handheld laser device was manually operated throughout the procedure.

All safety protocols were followed, including the use of protective eyewear by both patients and operators and equipment disinfection before and after each application.

### Standard postoperative care

All participants received the same standard postoperative care routinely provided after abdominoplasty. This included analgesic medication as prescribed by the surgical team, wound care and dressing management, recommendations for rest and gradual return to daily activities, and standardized physiotherapy instructions aimed at promoting mobility and preventing postoperative complications.

Importantly, these physiotherapy guidelines were part of the routine postoperative care protocol of the surgical service and were provided to both groups equally. Therefore, they were not considered an additional intervention within the context of this study.

Analgesic prescriptions followed the standard institutional postoperative protocol and were applied equally to both groups. Therefore, postoperative analgesic management was not expected to introduce systematic differences between groups.

### Saliva collection and biomarker analysis

Saliva samples were collected at three time points: T0 (1–6 h before surgery, in-hospital collection), T1 (48 h postoperatively, home collection), and T2 (7 days postoperatively, home collection).

Unstimulated saliva samples were collected using Salivette^®^ devices. Participants were instructed to collect samples in the morning while fasting and to avoid eating, drinking, or performing oral hygiene procedures for at least one hour prior to collection in order to minimize variability in salivary biomarker levels.

For T1 and T2, patients performed home collection using the Salivette^®^ kits. Samples were temporarily stored in home freezers and subsequently transported to the laboratory. A video call was conducted at each time point to verify correct sample collection and to assess pain using the Visual Analog Scale (VAS).

Upon arrival at the Cellular and Molecular Immunology Laboratory (UFCSPA), samples were stored at − 80 °C until biochemical analysis.

### Saliva deproteinization

To prepare samples for biochemical analysis, 500 µL of saliva were mixed with 500 µL of absolute ethanol at room temperature. The mixture was then centrifuged at 3,000 rpm for 10 min. Protein precipitation was achieved by centrifugation, and the resulting supernatant was collected and stored in microtubes at − 80 °C for subsequent assays.

### Cytokines (TNF-α and IL-10) analysis

TNF-α and IL-10 levels were quantified using ELISA kits (PeproTech/Thermo Fisher Scientific) according to the manufacturer’s instructions. Detection ranges were 23–3000 pg/mL.

### Nitrite quantification (Griess reaction)

Nitrite levels were measured as an indirect indicator of nitric oxide (NO) availability using the Griess reaction. For each well of a 96-well plate, 50 µL of deproteinized saliva were added along with 50 µL of Griess reagent, which consisted of 5% phosphoric acid (H₃PO₄), 2% sulfanilamide, 0.2% N-(1-naphthyl)ethylenediamine dihydrochloride (NED), and ultrapure water. The plate was incubated for 1 h at 37 °C, and absorbance was read at 540 nm using a spectrophotometer. Concentrations were determined based on a standard curve.

### Lipid peroxidation assay (MDA-TBARS test)

Lipid peroxidation was assessed using the MDA-TBARS method as described by Ohkawa et al., [21]. A total of 200 µL of deproteinized saliva were pipetted into separate test tubes. Each tube received 375 µL of acetic acid (2.5 M, pH 3.4), 375 µL of thiobarbituric acid (0.8%), and 500 µL of sodium dodecyl sulfate (8.1%). The reaction mixture was incubated in a water bath at 100 °C for 1 h, followed by centrifugation at 5,000 g for 15 min. The resulting supernatant was transferred to a 96-well plate and read at 532 nm using a microplate spectrophotometer. Final concentrations were calculated from absorbance values using a standard curve.

### Statistical analysis

Statistical analyses were performed using Python (version 3.12) with appropriate statistical libraries, including SciPy and StatsModels. Data were tested for normality using the Shapiro-Wilk test. For continuous variables, non-parametric comparisons between groups were performed using the Mann-Whitney U test. Repeated measures over time were analyzed using the Friedman test. Correlations were assessed using Spearman’s rank correlation coefficient. Multiple linear regression was used to assess predictors of biomarker levels (age, BMI, physical activity, surgical history). A significance level of *p* < 0.05 was adopted. In addition to traditional significance testing, effect sizes were calculated to quantify the magnitude of differences between groups and across time points. Between-group differences (PBM vs. Control) were assessed using Cohen’s *d*, while within-group changes across time points were evaluated with Cohen’s *dz* for paired samples. Effect sizes were interpreted according to conventional thresholds (small = 0.2, medium = 0.5, large = 0.8). This approach provides complementary information to *p*-values, allowing a more nuanced interpretation of clinical relevance beyond statistical significance.

## Results

A total of 31 patients completed the study, with 15 in the PBM group and 16 in the control group. The PBM group had a mean age of 46.8 ± 9.3 years, while the control group presented a similar age profile, with a mean age of 48.4 ± 8.5 years. The mean body mass index (BMI) was also comparable between groups, with 26.5 ± 2.5 kg/m² in the PBM group and 26.2 ± 2.0 kg/m² in the control group. Regarding ethnicity, 81.2% of participants in the PBM group and 86.7% in the control group self-identified as white. Tobacco exposure (current or former smoking) was reported by 18.8% of participants in the PBM group and 40.0% in the control group. The average length of follow-up with the surgical team was 25.3 ± 16.4 months in the PBM group and 27.3 ± 16.0 months in the control group.

No adverse events, complications, or PBM-related side effects were observed during the study period.

Pain perception was evaluated on postoperative days 2 and 7. On day 2 (T1), the PBM group reported a mean pain score of 2.7 ± 2.1, compared to 3.8 ± 2.7 in the control group. By day 7 (T2), mean pain levels had decreased in both groups, reaching 1.2 ± 1.3 in the PBM group and 1.4 ± 1.4 in the control group.

Descriptive statistics for each biomarker are presented in Table 1. Among the analyzed salivary biomarkers (TNF-α, IL-10, nitrite, and TBARS), no statistically significant changes were observed within groups over time for TNF-α, IL-10, or nitrite, nor between groups at any specific time point. However, a significant difference was detected for TBARS between the PBM and control groups at 48 h postoperatively (T1) (*p* = 0.034) in the unadjusted analysis. Baseline TBARS levels did not differ significantly between the PBM and control groups (Mann–Whitney U = 74.0, *p* = 0.072), although numerically higher values were observed in the PBM group. To account for this baseline variability, TBARS levels at T1 were further evaluated using a regression model adjusted for baseline TBARS (T0). In the adjusted model, baseline TBARS was a significant predictor of TBARS at T1 (β = 0.79, 95% CI: 0.52–1.07, *p* < 0.001), whereas the effect of PBM was not statistically significant (β = −0.053, 95% CI: −0.242 to 0.135, *p* = 0.567). Additionally, the change in TBARS from baseline to 48 h (ΔTBARS) did not differ between groups (Mann–Whitney U = 114.0, *p* = 0.828).

**Table 1:** Descriptive statistics for salivary biomarkers at baseline (T0), 48 h postoperatively (T1), and 7 days postoperatively (T2), for the photobiomodulation (PBM) and control groups

Biomarker	Timepoint	Group	Mean ± SD	Median [IQR]
TNF	T0	PBM (*n* = 15)	19.29 ± 8.35	14.35 [12.42–25.95]
TNF	T0	Control (*n* = 16)	20.45 ± 7.83	20.75 [12.74–22.79]
TNF	T1	PBM (*n* = 15)	18.10 ± 6.67	13.57 [12.65–23.12]
TNF	T1	Control (*n* = 16)	32.19 ± 54.66	19.09 [12.44–22.82]
TNF	T2	PBM (*n* = 15)	17.09 ± 6.48	12.58 [12.40–22.66]
TNF	T2	Control (*n* = 16)	18.26 ± 5.38	19.23 [12.61–21.53]
IL10	T0	PBM (*n* = 15)	6.13 ± 8.77	4.00 [3.60–4.39]
IL10	T0	Control (*n* = 16)	6.48 ± 10.07	3.77 [3.59–4.18]
IL10	T1	PBM (*n* = 15)	6.06 ± 8.82	3.76 [3.63–4.29]
IL10	T1	Control (*n* = 16)	7.22 ± 12.33	4.06 [3.54–4.69]
IL10	T2	PBM (*n* = 15)	9.04 ± 13.47	3.88 [3.49–4.38]
IL10	T2	Control (*n* = 16)	3.92 ± 0.67	3.68 [3.53–4.12]
Nitrite	T0	PBM (*n* = 15)	2.03 ± 1.77	1.67 [1.37–2.13]
Nitrite	T0	Control (*n* = 16)	2.99 ± 4.69	1.74 [1.31–2.54]
Nitrite	T1	PBM (*n* = 15)	2.36 ± 1.79	2.11 [1.81–2.28]
Nitrite	T1	Control (*n* = 16)	2.56 ± 1.77	2.30 [1.43–2.83]
Nitrite	T2	PBM (*n* = 15)	1.99 ± 0.77	2.08 [1.39–2.37]
Nitrite	T2	Control (*n* = 16)	2.05 ± 0.83	1.99 [1.37–2.45]
TBARS	T0	PBM (*n* = 15)	0.51 ± 0.45	0.23 [0.22–0.81]
TBARS	T0	Control (*n* = 16)	0.25 ± 0.11	0.22 [0.22–0.23]
TBARS	T1	PBM (*n* = 15)	0.50 ± 0.49	0.24 [0.22–0.61]
TBARS	T1	Control (*n* = 16)	0.24 ± 0.05	0.22 [0.22–0.23]
TBARS	T2	PBM (*n* = 15)	0.36 ± 0.40	0.22 [0.22–0.25]
TBARS	T2	Control (*n* = 16)	0.24 ± 0.07	0.22 [0.22–0.24]

*TNF* tumor necrosis factor alpha, *IL-10* interleukin-10, *TBARS* thiobarbituric acid reactive substances

Data are presented as mean ± standard deviation and median [interquartile range]

The Friedman test showed no significant temporal variation in TNF-α and IL-10 levels (*p* > 0.05). TBARS levels showed a small reduction from T0 to T1 in the PBM group (*p* < 0.05), although the magnitude of this change was small and between-group differences were not significant after baseline adjustment. Pain scores significantly decreased over time in both groups (*p* < 0.05).

Between groups (PBM vs. Control), effect sizes were generally small for all biomarkers across the three time points (Cohen’s d ranging from 0.04 to 0.36), supporting the absence of clinically relevant differences between groups. Within-group analysis showed that TBARS exhibited reductions of small magnitude between T0 and T1 in both groups (PBM: dz = − 0.04; Control: dz = − 0.11), indicating that the paired contrast alone did not demonstrate a consistent effect, despite the global significance detected by the Friedman test.

In contrast, postoperative pain showed large reductions between T1 and T2 in both groups (PBM: dz = − 1.25; Control: dz = − 1.32; overall effect dz = − 1.24), consistent with a robust clinical improvement over time. These findings indicate that salivary oxidative stress biomarkers showed only minor changes during the early postoperative period, while pain scores decreased substantially over time in both groups, consistent with the expected postoperative recovery trajectory.

### Correlation analysis

Spearman correlation analysis revealed no statistically significant associations between baseline biomarkers and postoperative pain scores (*p* > 0.05). However, some moderate, non-significant trends were observed. A moderate positive correlation was identified between TNF-α and IL-10 (*r* = 0.34; *p* = 0.061). Additionally, weak-to-moderate positive correlations were observed between TBARS and pain on postoperative day 2 (*r* = 0.24; *p* = 0.188) and on day 7 (*r* = 0.26; *p* = 0.151).

Pain scores on days 2 and 7 were significantly correlated with each other (*r* = 0.64; *p* < 0.001), suggesting consistency in individual pain perception throughout the postoperative recovery. Overall, these findings indicate that inflammatory and oxidative stress biomarkers at baseline are not strongly associated with short-term clinical outcomes such as postoperative pain.

No correction for multiple comparisons was applied due to the exploratory pilot design; therefore, findings with marginal p-values should be interpreted cautiously. These findings should therefore be interpreted as exploratory observations rather than confirmatory evidence of treatment effects.

### Multiple linear regression analysis

Multiple linear regression analyses were conducted to evaluate whether age, BMI, previous surgeries, bariatric surgery, and physical activity influenced salivary levels of TNF-α, IL-10, TBARS, and nitrite at baseline (T0).

For TNF-α, the model had low explanatory power (adjusted R² = −0.037), with no statistically significant predictors (*p* > 0.05). Although not statistically significant, BMI (β = 0.89; *p* = 0.225) and physical activity (β = 3.30; *p* = 0.361) showed positive trends.

The IL-10 model demonstrated an adjusted R² of − 0.139, and none of the predictors reached statistical significance. Age showed a weak and non-significant negative association (β = −0.15; *p* = 0.493).

For TBARS, the adjusted R² was − 0.043, again indicating limited explanatory value of the predictors. BMI (β = 0.05; *p* = 0.119) and age (β = −0.0067; *p* = 0.402) showed weak, non-significant trends.

In contrast, the model for nitrite yielded a higher adjusted R² (0.045), and physical activity was a statistically significant negative predictor (β = −3.09; *p* = 0.047). Participants who reported engaging in physical activity within the previous six months had significantly lower nitrite levels compared to sedentary individuals.

Overall, the models suggest that demographic and clinical variables had limited ability to explain the variance in inflammatory and oxidative stress markers. However, physical activity may play a role in modulating nitrite metabolism, potentially reflecting more efficient regulation of nitric oxide pathways.

## Discussion

Despite the growing interest in the effects of photobiomodulation on inflammatory and oxidative processes, studies investigating the relationship between salivary biomarkers and clinical outcomes in abdominal surgery remain scarce. Salivary biomarkers have emerged as a promising tool for monitoring postoperative recovery, as they allow for the simultaneous assessment of local and systemic inflammatory responses. Their application has been well documented in oral and maxillofacial surgery, where salivary biomarkers provide insight into tissue healing and inflammatory modulation [27]. Furthermore, the non-invasive nature of saliva collection makes this approach particularly advantageous in populations for whom blood sampling may be challenging, such as pediatric or post-surgical patients with limited venous access [22]. This underscores the novelty and clinical relevance of the present study, as one of the few investigations applying salivary biomarker analysis to monitor recovery in abdominal aesthetic surgery.

Baseline TNF-α levels in both intervention and control groups were within or slightly below the reference ranges reported for healthy adults, indicating that participants were not markedly pro-inflammatory at baseline. Reported values in the literature vary considerably (22–47 pg/mL), reflecting methodological and population differences [6, 30]. In our study, mean TNF-α levels were at the lower end of this spectrum, supporting the absence of heightened inflammation preoperatively. The higher variability observed in the PBM group at the intermediate timepoint suggests heterogeneous individual responses to PBM. TNF-α values decreased slightly over time in both groups but remained within expected ranges, consistent with a normal resolution of inflammation after surgery. No significant between-group differences were observed.

IL-10, a key anti-inflammatory cytokine, presented elevated mean values across both groups, with consistently higher levels in the PBM group. This pattern suggests a postoperative compensatory response to surgical trauma, potentially enhanced by PBM. IL-10 is fundamental for regulating inflammation and promoting tissue repair [35, 36]. Previous reports indicate that PBM may stimulate IL-10 release, reducing inflammation and accelerating recovery [1]. In our study, however, no significant between-group differences were detected, possibly due to individual variability or limited PBM exposure. The wide dispersion of IL-10 values, particularly in the PBM group (IL-10 C = 12.87 ± 23.59 pg/mL), supports the notion of heterogeneous responses influenced by baseline inflammatory status or metabolic factors.

Although TNF-α and IL-10 values fluctuated within ranges considered normal for healthy adults, small changes may still reflect subtle immune modulation following abdominoplasty. However, the clinical sensitivity of these markers in detecting acute surgical inflammation requires further validation. Given that both TNF-α and IL-10 remained within normal ranges, their usefulness as salivary biomarkers for acute postsurgical monitoring may be limited, underscoring the need for cross-validation with serum levels in future studies.

In the present study, TBARS levels showed a reduction at 48 h postoperatively in the PBM group. However, this finding should be interpreted with caution. Although TBARS showed a statistically significant reduction at T1 in the unadjusted analysis, the very small effect size and the absence of significance after baseline adjustment indicate that this result should be interpreted carefully. This pattern may reflect biological variability as well as the exploratory nature of this pilot study. Nevertheless, the observed reduction in TBARS may be consistent with the antioxidant effects reported for PBM in experimental studies. Animal models of diabetes, arthritis, and high-intensity exercise have shown that PBM can reduce lipid peroxidation and enhance antioxidant defenses such as SOD, CAT, and GPx activities [12, 33]. Despite these findings, variability in TBARS assays and differences in PBM parameters, such as wavelength, energy density, and irradiation protocols, may contribute to heterogeneous outcomes across studies [32].

Although a statistically significant reduction in TBARS was observed, the clinical significance of this finding remains uncertain, as minimal clinically important difference (MCID) thresholds for salivary oxidative markers have not yet been established. Further clinical trials with standardized PBM protocols and larger sample sizes are needed to clarify the potential antioxidant effects of PBM during postoperative recovery.

Nitrite levels remained within reference ranges in both groups, with no significant temporal variation. Regression analysis revealed, however, that participants reporting regular physical activity in the preceding six months had significantly lower baseline nitrite levels (β = − 3.09; *p* = 0.047). This finding supports the hypothesis that exercise modulates nitric oxide metabolism by upregulating eNOS, enhancing vasodilation, and preserving vascular health [3, 39]. Physical activity also reduces oxidative stress, thereby maintaining NO bioavailability [5, 19]. The lower nitrite values in active participants may reflect more efficient NO turnover and reduced nitrite accumulation, consistent with cardiovascular benefits of regular exercise.

Although between-group differences did not reach clinical relevance (Cohen’s *d* = 0.04–0.36), this finding supports the interpretation that PBM and control groups exhibited largely comparable biomarker trajectories. The within-group reductions in TBARS showed small effect sizes, suggesting that although statistically significant, the magnitude of oxidative stress modulation was modest.

The use of saliva as a biological matrix for monitoring postoperative biomarkers also deserves consideration. Salivary biomarkers have increasingly been investigated as non-invasive indicators of systemic inflammation. Several circulating mediators can diffuse into saliva through transcellular or paracellular mechanisms in the salivary glands. Previous studies have demonstrated correlations between salivary and serum levels of inflammatory mediators and oxidative stress markers in various systemic conditions. However, most investigations have focused on oral or systemic diseases rather than postoperative responses following non-oral surgeries. Although saliva offers practical advantages such as non-invasive collection and suitability for repeated measurements, its sensitivity for detecting inflammatory changes after abdominal surgery remains uncertain. Salivary biomarkers may also be influenced by salivary flow rate, oral health status, and individual biological variability [15, 17, 9].

Pain intensity decreased significantly in both groups from T1 to T2. However, the PBM group consistently exhibited numerically lower pain scores, although this numerical difference did not translate into statistically significant between-group differences. Pain scores at both timepoints were strongly correlated (*r* = 0.64; *p* < 0.001), indicating stable perception during early recovery. The analgesic effects of PBM have been attributed to several biological mechanisms. These include modulation of inflammatory mediators, reduction of reactive oxygen species, stimulation of mitochondrial ATP production, promotion of angiogenesis and lymphatic drainage, and modulation of nociceptive pathways with endogenous opioid release []. Clinical studies in oral and maxillofacial procedures also support PBM as an adjunctive strategy for reducing postoperative pain, swelling, and functional impairment [2, 7, 13]. Variability in PBM parameters, however, remains a limitation for protocol standardization [8, 14, 24]. In contrast, postoperative pain reduction demonstrated large effect sizes in both groups (dz ≈ − 1.25), supporting its clinical relevance regardless of group allocation. This large within-group effect indicates a clinically meaningful reduction in pain, even in the absence of significant between-group differences.

While most PBM protocols in the literature involve multiple sessions, our study applied only a single session prior to discharge, mainly due to clinical feasibility in a surgical setting. This choice may have limited the potential cumulative or sustained effects of PBM. However, some clinical evidence suggests that a single session can also produce meaningful postoperative outcomes. For instance, Yüksek et al. [40] reported comparable effects between single and repeated sessions for pain, edema, and trismus after third molar extraction. Similarly, Tenore et al., [38] demonstrated significant pain reduction on days 0, 1, and 7 following a single intraoral and extraoral application. Roccon et al., [31] also found a single PBM session effective for symptom relief in Oral Lichen Planus, with an excellent safety profile. These findings support the rationale of testing a single-session approach in abdominoplasty, while future studies should evaluate repeated applications to better establish dose–response relationships and sustained benefits.

In addition to the use of a single PBM session, the energy delivered per irradiation site (3 J) may have represented a conservative dosing strategy for treating a large surgical area during a single postoperative session. This dose was selected to allow complete coverage of the surgical area within a clinically feasible treatment time before hospital discharge. It is possible that higher energies per irradiation site or repeated PBM sessions could produce more pronounced modulation of postoperative inflammatory and oxidative biomarkers. Therefore, future dose–response studies evaluating different energy levels and treatment schedules are warranted to determine the optimal PBM parameters for postoperative recovery.

The relationship between inflammatory/oxidative markers and postoperative pain perception is complex and multifactorial, involving both peripheral and central nociceptive mechanisms. In our study, Spearman correlations between inflammatory/oxidative markers and pain scores revealed no statistically significant associations, although moderate trends were observed for TNF vs. IL-10 and TBARS vs. pain. These trends suggest that inflammatory markers alone may not fully explain perceived pain or recovery dynamics. TBARS levels showed a non-significant trend toward association with pain scores, consistent with the hypothesis that oxidative stress may contribute to postoperative discomfort. The absence of statistically significant correlations may be related to the relatively small sample size and interindividual variability in inflammatory responses. Additional confounding factors, such as anesthetic regimen, analgesic protocols, and baseline health status, may also have influenced these results. Surgical trauma is known to increase oxidative stress through tissue injury, ischemia–reperfusion processes, and inflammatory activation, which may explain the relevance of monitoring lipid peroxidation markers such as TBARS during postoperative recovery [23].

Inflammation and oxidative stress are biologically plausible contributors to postoperative pain. However, their impact is also influenced by metabolic status, surgical technique, pharmacological management, and psychosocial factors. The lack of significant associations in this dataset highlights the multifactorial nature of postoperative pain and underscores the need for integrative approaches in future research. Studies with larger samples, multimodal biomarker panels, and standardized analgesic regimens will be essential to clarify mechanistic links between inflammation, oxidative stress, and pain in surgical recovery.

Taken together, these findings suggest that PBM may influence oxidative and inflammatory pathways during postoperative recovery. However, the magnitude and clinical relevance of these effects remain uncertain in the present pilot study.

Several methodological limitations must be acknowledged. The small sample size (*n* = 31) may have limited statistical power to detect subtle between-group differences or biomarker–pain associations. Additionally, the non-randomized design introduces potential selection bias, which may have influenced group comparability. Future randomized controlled trials are needed to minimize this source of bias. Biological variability, including age, comorbidities, and individual recovery responses, may have influenced biomarker levels and pain perception. The absence of a sham-irradiated control group is a limitation, as placebo effects cannot be excluded. Future trials should incorporate sham PBM to minimize bias. The three assessment timepoints may not fully capture the dynamic fluctuations in inflammatory and oxidative markers following surgery. Furthermore, variability in PBM application parameters and the limited number of sessions could have influenced treatment effects. Salivary biomarkers were not validated against serum levels in this study, which limits conclusions regarding their ability to fully reflect systemic inflammatory responses following non-oral surgical procedures such as abdominoplasty. Although saliva represents a convenient and non-invasive biological matrix, its biomarker concentrations may be influenced by local oral conditions, salivary flow rate, and individual biological variability [20, 26, 34]. Additionally, patient-reported outcomes were limited to pain assessment using the Visual Analog Scale. Other relevant dimensions of postoperative recovery, such as quality of life, patient satisfaction, functional recovery, and perceived wound healing, were not evaluated and should be considered in future studies. Additionally, the use of a single PBM session—while clinically pragmatic—may have limited the potential cumulative effects typically observed with repeated applications. Lack of prospective trial registration also limits transparency and reproducibility. To strengthen the evidence base, future studies should include randomized controlled trials with larger cohorts and standardized PBM protocols. Extended biomarker monitoring and validation of salivary biomarkers against serum measurements will also be important to clarify the clinical and mechanistic role of PBM in surgical recovery. No correction for multiple comparisons was applied due to the pilot design; therefore, marginal p-values should be interpreted with caution. This decision was maintained due to the exploratory nature of this pilot study, which aimed to identify preliminary trends to guide future controlled trials with larger samples and more robust statistical adjustment.

## Conclusion

Overall, these preliminary findings suggest that PBM appears to be a safe intervention and may influence oxidative and inflammatory pathways during postoperative recovery after abdominoplasty. However, given the small sample size and methodological limitations of this pilot study, these findings should be interpreted as hypothesis-generating rather than definitive evidence of clinical benefit.

Future randomized controlled trials with sham-irradiated controls, prospective registration, validation of salivary biomarkers against serum levels, and investigation of minimal clinically important difference (MCID) thresholds are needed to clarify the clinical relevance and reproducibility of PBM effects in surgical recovery.

## Data Availability

The datasets generated and/or analyzed during the current study are not publicly available due to privacy and ethical restrictions involving patient data. However, they are available from the corresponding author upon reasonable request and with appropriate institutional approvals.
